# Biomechanical Model of Non-Contact Anterior Cruciate Ligament Injury Concerning Shin Angle and Field Surface Traction Parameters—With a Piezo2 Interpretation

**DOI:** 10.3390/sports13120414

**Published:** 2025-11-21

**Authors:** Tekla Sümegi, Balázs Sonkodi, Krisztián Havanecz, István Berkes, Bence Kopper

**Affiliations:** 1 Faculty of Kinesiology, Hungarian University of Sports Science, 1123 Budapest, Hungary; 2 Hungarian National Academy of Handball, 8630 Balatonboglár, Hungary; 3 Department of Health Sciences and Sport Medicine, Hungarian University of Sports Science, 1123 Budapest, Hungary; 4 Department of Sport Medicine, Semmelweis University, 1122 Budapest, Hungary; 5 Faculty of Health Sciences, Institute of Physiotherapy and Sport Science, University of Pécs, 7622 Pécs, Hungary; 6 Physical Activity Research Group, Szentágothai Research Center, University of Pécs, 7622 Pécs, Hungary; 7 Training Theory and Methodology Research Center, Hungarian University of Sports Science, 1123 Budapest, Hungary

**Keywords:** anterior cruciate ligament (ACL) injury, MRI images, biomechanical factors, knee joint load, surface characteristics, acquired Piezo2 channelopathy

## Abstract

Background: Biomechanical factors behind non-contact anterior cruciate ligament (ACL) injury in soccer and handball are still not fully understood. Unfortunately, ACL injuries more frequently appear in game situations. Aim: To describe a possible ACL injury mechanism in male professional handball players using MRI images and our own biomechanical model. Hypothesis: The friction parameters of the surface have extreme importance in the non-contact ACL injury mechanism. If the surface is more slippery, the horizontal component of the ground reaction force (GRF) will be smaller, consequently the torque originating from the GRF acting on the knee will be greater during the landing phase of a vertical jump, resulting in greater abduction effect on the knee. Consequently, the risk of knee injury increases. Methods: We have collected MRI images and anthropometric data of 15 healthy male individuals (age 19–23) to create a biomechanical model to calculate the torques in the knee to obtain more knowledge about ACL injury mechanism. Results: The lower extremity lean angle during the landing phase of a jump and friction parameters substantially affect abduction torques in the knee and consequently the risk of ACL injury occurrence. Conclusions: The landing posture when the knee is fully extended during landing is highly unfortunate for the ACL, compared to when the knee is partially flexed. If the knee is fully extended, greater hip abduction will increase the risk of an ACL injury, and if the surface is more slippery, e.g., the surface is wet, the possibility of ACL injury is even greater. In addition, we also applied a molecular working hypothesis through acquired Piezo2 channelopathy theory, as the proposed preceding neuromuscular disruptor prior to non-contact ACL injury.

## 1. Introduction

One of the most feared injury types among athletes is the anterior cruciate ligament (ACL) tear. In many cases this injury results in the end of the athlete’s career, especially in soccer or handball. This type of injury is impossible to predict, the rehabilitation is long and costly [1] and the probability of re-injury occurrence is extremely high [2,3,4]. The importance of the subject is emphasized by the significant number of articles on ACL tear with many theories explaining the injury phenomenon [5,6,7,8,9,10,11]. Universally two types of ACL injuries are being distinguished in the literature, contact and non-contact injuries. The majority of sports-related ACL injuries happen in non-contact scenarios, typically involving landing, rapid deceleration, or sudden change in direction [12]; therefore in this paper, we focus on non-contact ACL injuries.

After the inception of the neurocentric bi-phasic injury mechanism of non-contact ACL injury theory [11], Boden and Sheenan also stressed the relevance of preceding minor perturbation, leading to disruption of the neuromuscular system, prior to ACL injury [13]. Later, even the microdamage of an ion channel was hypothesized for the initiation of the primary damage of this bi-phasic non-contact injury mechanism, namely in the form of an acquired Piezo2 channelopathy on the proprioceptive somatosensory nerve terminals [14]. Hereafter, the current paper relies on this preceding acquired acute Piezo2 channelopathy as a working hypothesis that may explain the disruption of the neuromuscular system and consequently expose the ACL to increased injury risk.

Biomechanically, it is universally accepted that the primary cause of ACL tear is the mechanical overload of the ligament [5,10]. There are numerous biomechanical factors which influence the overload of the ligament, such as extreme force in the quadriceps muscle, extreme ground reaction force (GRF), small knee flexion while landing, intercondylar notch impingement, axial compressive forces on the lateral aspect of the joint, non-optimal hamstring to quadriceps (H:Q) strength ratio as a predictor of ACL injury and knee valgus (knees collapsing inward), poor quadriceps control and unstable landing (which can be monitored with the Tuck Jump exercise) [5,10,15,16,17].

One of the most frequently occurring mechanisms behind ACL injuries is the rotation-pivoting movement of the tibia around its longitudinal axis [10]. Meanwhile a great number of studies concluded that the anterior shear force at the proximal end of the tibia incorporated with knee valgus, varus and internal rotation moments results in a pronounced ACL load [8,18,19,20]. Moreover, a pilot case study showed that the point of attack of the resultant force in the tibia skewed toward the medial proximal tibia during landing [21]. DeMorat et al. demonstrated the effect of extreme (greater than 4500 N quadriceps force) quadriceps muscle generated anterior shear force at the proximal end of the tibia through the patella tendon on ACL ruptures [22]. Quadriceps contraction also increases the compressive loads on the tibiofemoral joint, thus contributing to ACL injury [23]. Additionally, if the knee flexion angle decreases during athletic tasks, the anterior shear force increases as it is further exacerbated by higher quadriceps force, which increases the patella tendon–tibia shaft angle, thereby generating more shear force on the tibia [10].

Knee flexion and abduction angles during the landing phase of a vertical jump play a crucial role in ACL injury prevention. Hewett et al. revealed that ACL-injured athletes had significantly greater knee abduction angles (8.4°) at landing and higher peak external knee valgus moments, as well as higher peak vertical GRF compared to uninjured athletes. Additionally, significant correlations between knee abduction angle and peak vertical GRF were identified in the injured group. The maximum knee flexion angle at landing was 10.5° less in the ACL injured group. Excessive valgus torques on the knee can increase anterior tibial translation and ACL load; namely, if an athlete lands in a dynamic valgus or with unusual foot placement, the likelihood of ACL injury increases [8,10,24,25]. Another video analysis-based study presented that the distances from the center of mass (COM) to the point of contact were greater and consequently the angles between the thigh and vertical axis were greater when ACL rupture occurred compared with non-injured landings [26,27].

In a sport-specific movement, like landing or deceleration, GRFs are always present. The foot, ankle, knee, hip and muscles around the joints help absorb the reaction forces from the upper body and the GRFs [5]. Soccer- and handball-specific jumps often differ from double-legged landing actions in other sports as many in game scenarios such as cutting, passing, or landing after a header require athletes to support their full body weight on a single limb [28]. Kamari et al. have observed greater GRFs, increased knee valgus, and reduced knee flexion at initial contact (IC) during a single leg drop landing compared to a double leg drop landing [12]. Boden and colleagues concluded through video analysis that individuals who sustained ACL injury reached the ground with a flat foot or with heels, while control subjects landed on their forefoot [15]. They also concluded that the plantarflexed ankle helps dissipate the forces during landing, thus protecting the ACL. This is important because when landing with one leg, the peak vertical GRF could reach five to even eighteen times the body weight and as ACL ultimate tensile strength is limited (approximately 2300 N [29]), landing on a forefoot and using the dampening effect of the ankle is a key aspect in preventing ACL rupture.

By viewing numerous video recordings of ACL injuries of soccer players during match situations, we have concluded that another mechanism with very specific parameters might also be responsible for the frequent occurrence of the injury, especially concerning soccer players. In many cases the injury occurs in the landing phase, when executing a jump in the final phase, the foot contacts the ground. When analyzing the movements of the athletes in the cases that we have observed, before the injury at landing pivoting of the knee, namely rotation of the tibia around its longitudinal axis, was not distinguishable. However, in these scenarios, immediately before contact with the surface, the knee was in an extended and the thigh was in an abducted position viewed from the frontal plane. At IC, when deceleration of the body would occur, sideways slippage (accidental mishap) of the foot was visible as viewed from the frontal plane, away from the body followed by abnormal abduction of the knee joint and the collapse of the athlete, universally distinguished in the literature as knee valgus collapse. We hypothesize that if this type of movement pattern is recognizable, then the traction parameters of the soccer field or the handball court could influence ACL injury incidence probability. Our hypothesis is highlighted by empirical observations that non-contact ACL injuries are more frequently observed in game situations—when the soccer field is usually being treated extensively with water to create a more slippery surface to increase the speed of the game—and very rarely at trainings. On the contrary, during handball games, if possible, the surface is thoroughly wiped after a player’s fall to decrease the possibility of slippage on a wet spot. McMaster had also highlighted that athletes may be at the greatest risk for non-contact ACL injury during games and especially towards the end of half-time, at the finishing of games or at season end [30]. Our preceding primary damage working hypothesis further comprises that the aforementioned excitatory Piezo2 ion channels are not only stretch and force gated [31], but may be an ultradian sensor [32] as well. Therefore, the slippery surface may not only increase axial compression forces and in response a point of attack of the resultant force in the medial proximal tibia during landing, but induce over-excessive stretch (mimicking an over-excessive eccentric contraction) and resultant contribution to the aforementioned theorized microdamage of Piezo2 (failure to functionally activate abruptly in an ultrafast fashion).

To investigate the complex rationale discussed previously, our aim is to introduce a simplified biomechanical model to examine the different contributing factors occurring in this type of ACL injury. The above-introduced injury mechanism, body position, lower extremity position might sound unnatural, but there are some influencing factors that should be taken into consideration. Jumps during a sport event are fundamentally different compared with vertical jumps examined in a laboratory environment using 3D movement analyzing systems [33]. In a game situation the landing is almost never symmetrical for the two legs either concerning the position, or the distribution of GRF for the two legs. In the movement of the COM, the trajectory of the movement is almost never vertical; usually, there is a horizontal element, and consequently at the landing phase the athlete’s body possesses a horizontal velocity component. We must also consider the surrounding elements, as contrary to an isolated laboratory environment, many external inputs afferent signals of proprioceptors must be processed by the central nervous system, and consequently optimal landing posture might not always be achievable.

## 2. Materials and Methods

In order to acquire the required anthropometric values of the knee that were used for the mathematical model, we analyzed the geometric parameters of the MRI images of athletes that had been previously recorded by medical professional personnel in a Hungarian medical facility (Kaposi Mór Oktató Kórház-Kaposvar city medical Hospital, Kaposvár, Hungary). The individuals were athletes of the Hungarian Handball Academy (NEKA) with signed consent documents to approve the evaluation of the recorded data. We used MRI pictures of the dominant leg of the athletes, where inclusion criteria were no prior injury occurrence in the limb. From the acquired images, we have only determined the required geometric values, and no medical evaluation was executed. The study was approved by the Science Ethics Committee of the Hungarian University of Sports Science, Ethical Approval Number: TE-KEB/18/2022.

**Figure 1:**
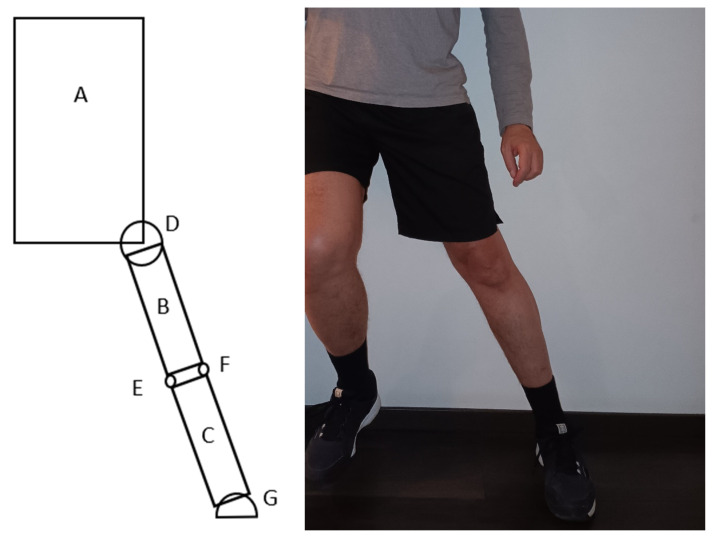
Model of an athlete in the frontal plane viewed from the front in the moment of landing on the surface with one leg (**left**), the athlete for reference (**right**). Objects in the model: A—trunk; B—thigh; C—shin; D—hip joint; E—medial condyle of the knee; F—lateral condyle of the knee; G—ankle and foot.

To understand the biomechanical properties of the mechanism that we introduce in this paper, we will use a simplified model of the human body. In our model the following objects represent the body elements: A—trunk; B—thigh; C—shin; D—hip joint; E—medial condyle of the knee; F—lateral condyle of the knee; and G—ankle and foot (Figure 1). In Figure 1 our model in the left drawing represents the athlete in the frontal plane observed from the front, at the beginning of contact with the surface in the landing phase of a jump. In Figure 1 on the right the real athlete is visible taken as a schematic reference for the drawing. It is important to highlight that based on postures, we have observed in numerous video recordings the thigh is in an abducted position and the knee is fully extended. The other leg is not visible in the left drawing in Figure 1, as that leg only makes contact after the indicated leg—where the ACL injury would occur—and consequently does not have a significant role in the injury mechanism that we discuss.

**Figure 2:**
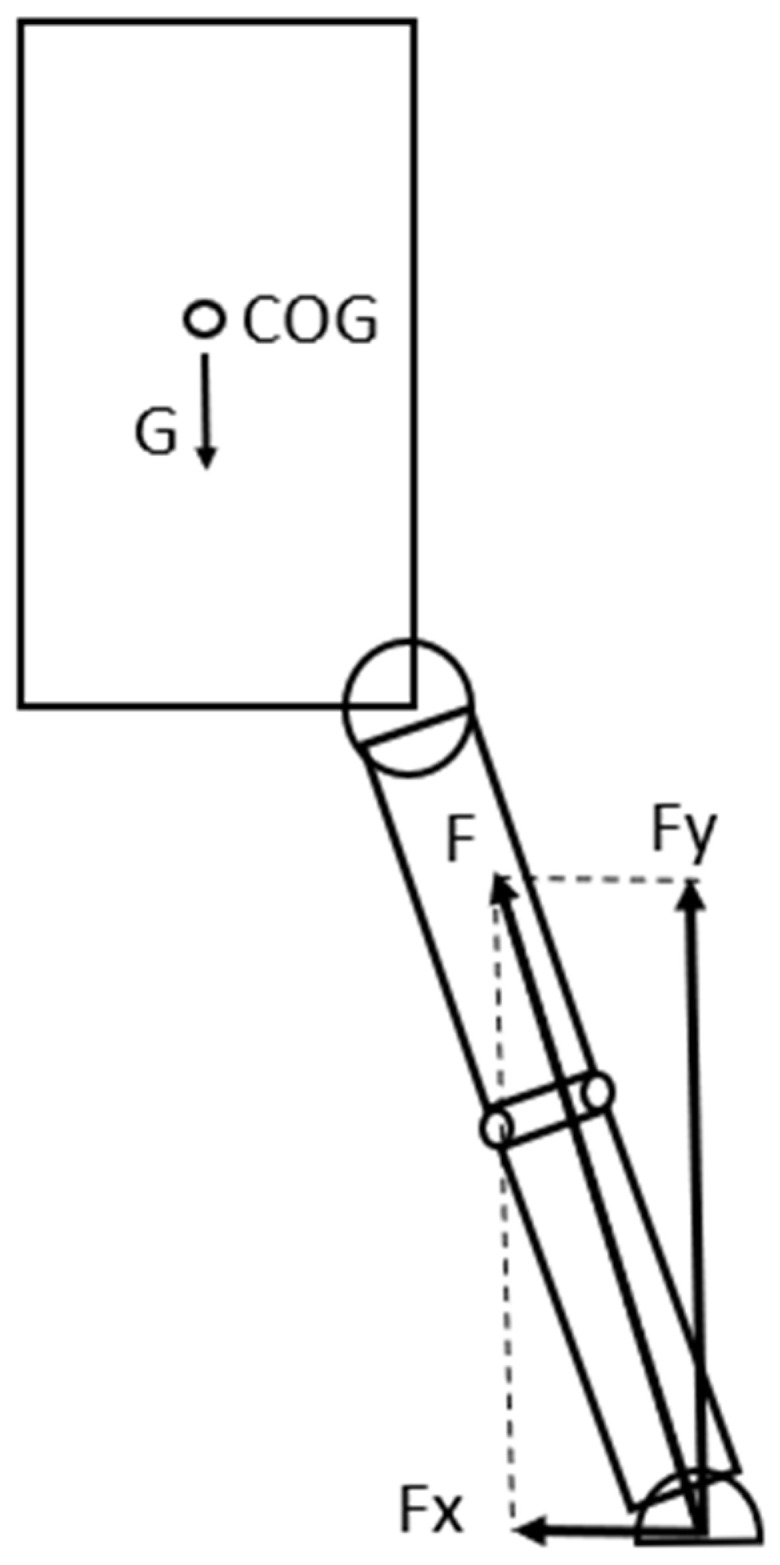
External forces acting on the athlete in the model when contact with the surface occurs after a jump: G—gravitational force; F—ground reaction force; Fx—horizontal component, Fy—vertical component of the ground reaction force.

There are two major external forces acting on the athlete in this situation: the gravitational force—G—acting in the COG (Center of Gravity—technically similar to COM) for the individual and the GRF. For the purpose of our later analysis, the GRF can be split into horizontal—Fx—and vertical—Fy—components (Figure 2) (This set-up of the acting forces is universally used in sport movement analysis). After an execution of a vertical jump in the landing phase, the net GRF can be 5–10 times greater or more than the gravitational force.

**Figure 3:**
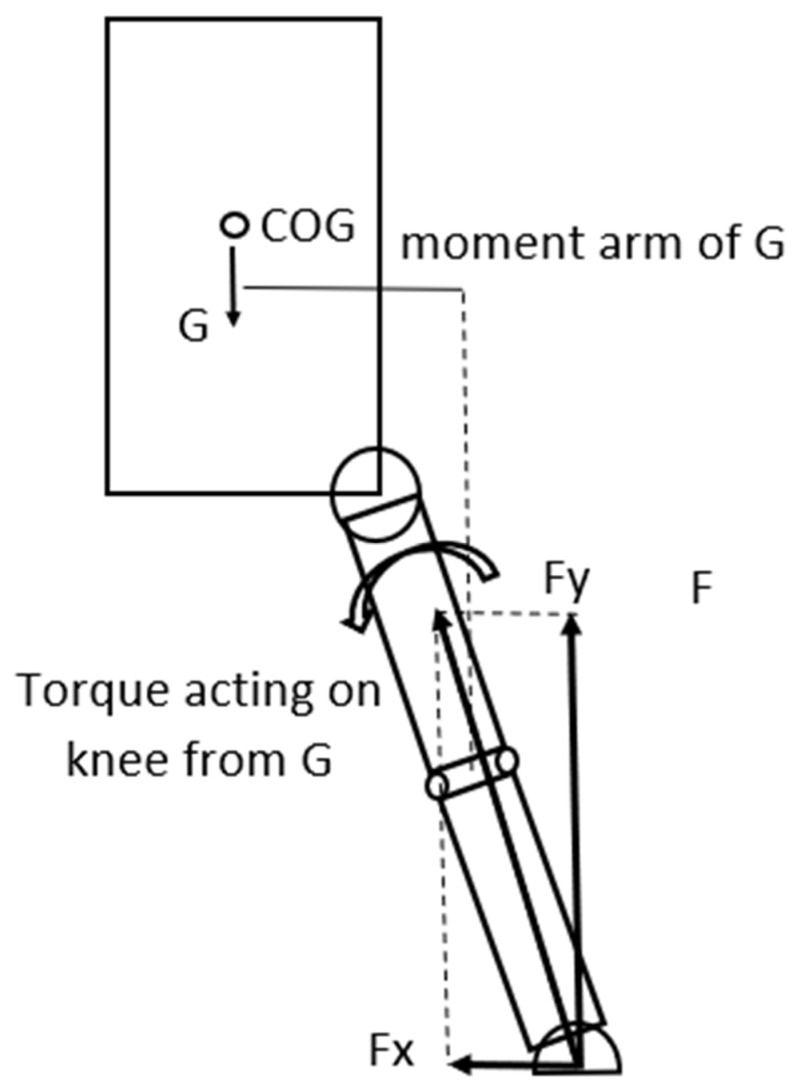
The gravitational force acting on the COG creates a torque around the knee that rotates the trunk-hip system in a counter-clockwise direction around it.

By using our model, we can identify a torque acting on the knee from the gravitational force that is trying to rotate the trunk and the thigh around the knee in a counter-clockwise direction in the frontal plane (Figure 3). If we analyze the model in detail, based on the structure of the knee and considering that the knee is in an extended position in the model, we can conclude that the torque originating from the gravitational force is trying to rotate the thigh around the medial condyle of the knee joint. In this position the joint surfaces—the medial and lateral condyles act as axes for the knee joint and around these axes, minimal abduction and adduction of the knee can occur. As Fx is present, the orientation of the GRF is not vertical but pointing more-or less towards the knee, consequently the torque from the GRF acting on the knee can be considered minimal (Figure 3). In this case the load on the ACL is not extreme; therefore, injury is not expected to happen.

**Figure 4:**
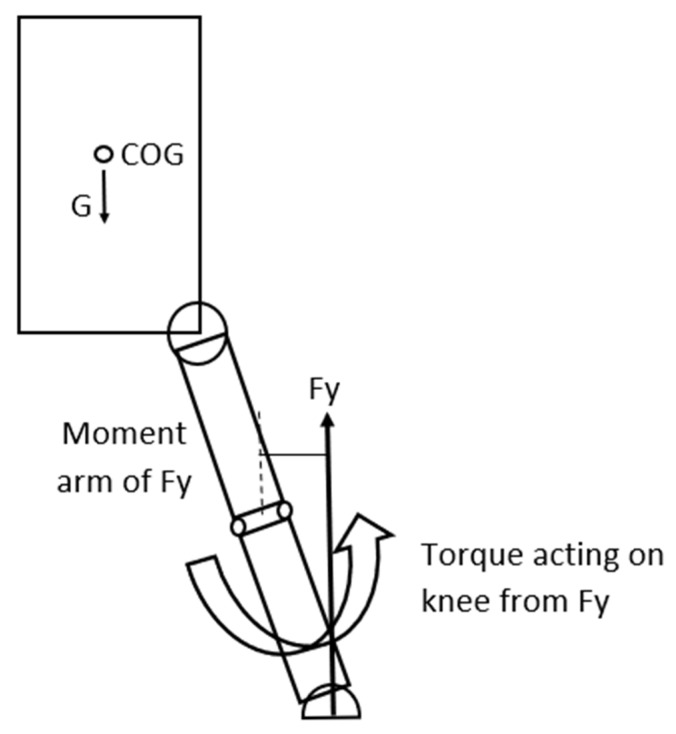
The GRF creates a torque around the knee that rotates the shin in a counter-clockwise direction increasing abduction.

**Figure 5:**
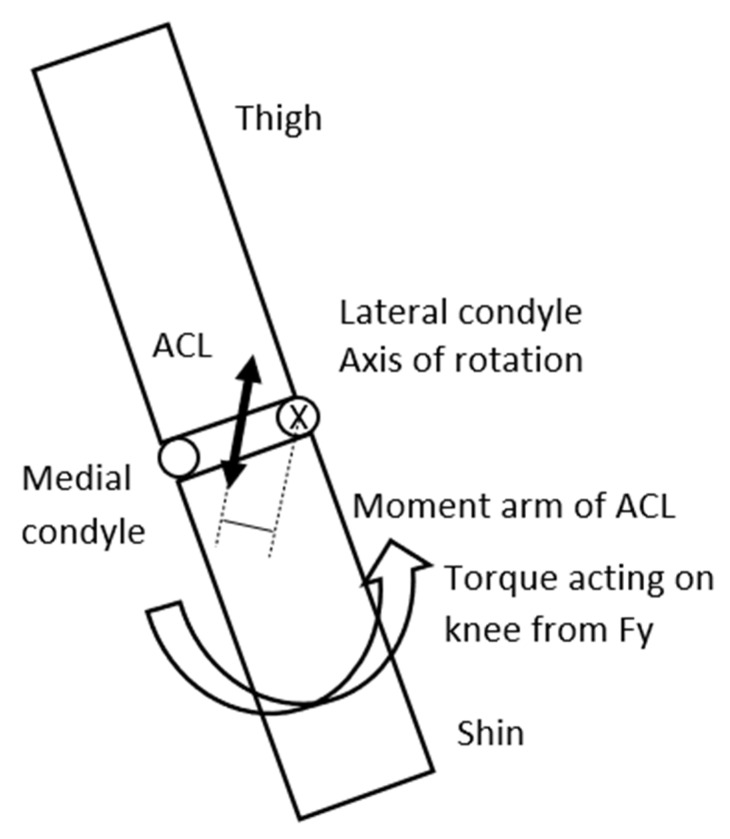
Shin and thigh viewed in the frontal plane from the front. To partially counter the torque and rotating effect of the GRF around the lateral condyle of the knee—that is the axis of rotation, tension is developing in the ACL that will generate a torque in the reverse direction.

In this landing situation, if the surface is slippery then it significantly affects the load on the knee. In a slippery surface the horizontal component of the GRF is minimal, or if the foot of the athlete slips or slides sideways at contact, this horizontal force component (Fx) can even be zero. However, it must be emphasized that this is a theoretical boundary condition in a simplified model. Experimental data acquisition is required in the future in order to improve it into a more universally usable model. Consequently, if Fx is zero only the vertical component of the gravitational force (Fy) is present. Fy will create—as the Fy can be 5–10 times greater, or more than the gravitational force, (partially because as we have observed in the video recordings, in the cases the knee is in an almost entirely extended position)—a significant torque acting on the knee. This torque will try to rotate the shin around the knee in a clockwise direction (Figure 4). As Fy is significantly greater than G—especially if the knee is in an extended position—the torque originating from the GRF can also be significantly greater acting on the knee. This torque will rotate the shin around the lateral condyle of the knee joint, consequently generating significant load on the ACL. In an extreme situation this biomechanical load can exceed the mechanical tensile strength of the ACL, consequently injury will occur in the ligament [32] (Figure 4 and Figure 5). Nevertheless, we must emphasize three influencing factors. Firstly, torque generation in the human body is not a binary process, although in this model, considering the conservative approach, we have used the maximum values we have found in the literature. Secondly, another aspect we must consider is that this simplified model is not able to take into account the inter-individual variability in ligament properties. Mechanical tensile strength is individually different from person to person; therefore, the tensile threshold used in the calculation to determine injury occurrence is subjective and can be considered as a limitation. Furthermore, neural activation and neuromuscular compensation differ from person to person, which is also an influencing factor in the properties of the active components.

There is a passive and an active mechanism that can decrease the abduction of the knee and therefore the stress on the ACL in this model. The passive tension originating from the medial collateral ligament (MCL) and the active tension originating from the quadriceps pulling the tibia through the quadriceps–patella–patellar tendon towards the femur, the semitendinosus–semimembranosus also pulling the tibia towards the femur, and the gastrocnemius medialis pulling the femur towards the tibia. We have assumed in the calculations that muscles are completely activated to determine the tensile threshold where injury occurs. Naturally if the muscle activation is limited, then the tensile stress will be greater on the ACL. As the knee rotates around the lateral condyle previously defined as the axle of abduction, the MCL will stretch and can take over some of the load on the ACL. This theory is supported by the fact that in many cases when ACL tear happens, MCL damage can also be observed [34]. However, when the ACL completely tears, in many cases the MCL will partially remain intact, therefore it can be speculated that the load transfer from the ACL to the MCL is limited. The ultimate tensile strength of the MCL according to a study is 799 ± 209 N [35].

**Figure 6:**
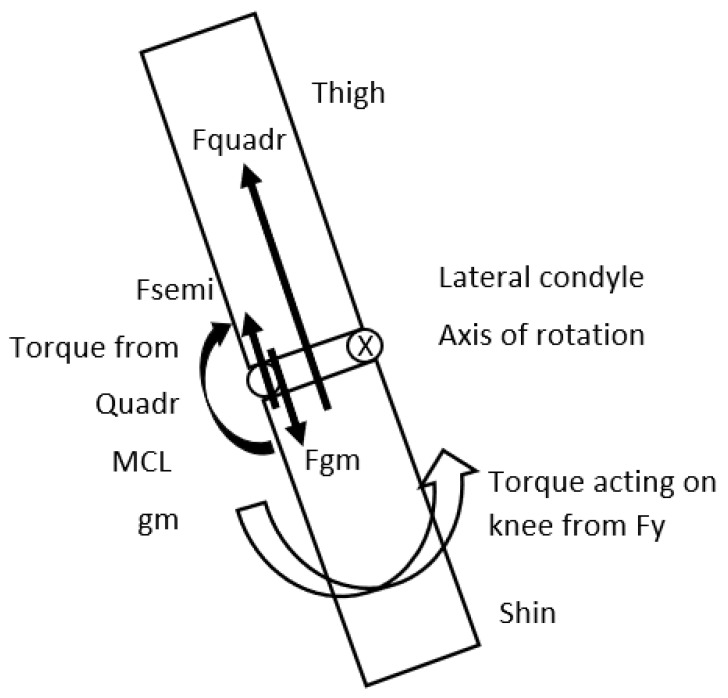
Shin and thigh viewed in the frontal plane. Fy (vertical component of the GRF) will generate a torque to rotate the shin around the lateral condyle of the knee creating abduction. The force of the quadriceps—Fquadr; semitendinosus, semimembranosus force—Fsemi, force of the Gastrocnemius Medialis—Fgm and the stretch of the MCL will reduce this torque, creating an adduction effect, consequently decreasing load on the ACL.

The activation level and consequently the tension in the quadriceps, semitendinosus–semimembranosus complex, soleus and the gastrocnemius medialis significantly affect ACL load, because if the knee is in an extended position, torque as a result of extreme Fy will act on the knee and generate an abduction effect, and consequently rotation will occur around the lateral condyle, and the tension of these muscles will decrease the torque of Fy based on the anatomical structure of the knee and decrease abduction [36] (Figure 6).

## 3. Results

To acquire more insight into the injury mechanism that we have discussed and introduced above, we have created a mathematical model to calculate the torques in the knee. No previous specific statistical software was used to evaluate the model. We must highlight that although some parameters could be measured directly, for some other parameters we had to rely on previous research results from different sources (Table 1 and Table 2). In some cases, we decided to use the maximum values available in the literature to draw a conclusion while maintaining a conservative approach. For other anatomical values that we could not find in the literature, we have processed previously recorded MRI images of athletes.

Factors responsible for the torque generating abduction around the lateral condyle: GRF at landing—GRF-lateral condyle moment arm. Factors responsible for the torque generating adduction around the lateral condyle: ACL stress—ACL-lateral condyle moment arm; MCL stress—MCL-lateral condyle moment arm; patellar tendon (PT)/quadriceps stress—patellar tendon-lateral condyle moment arm; semitendinosus stress (SEMIT)—semitendinosus-lateral condyle moment arm; bodyweight—COG-lateral condyle moment arm.

**Figure 7:**
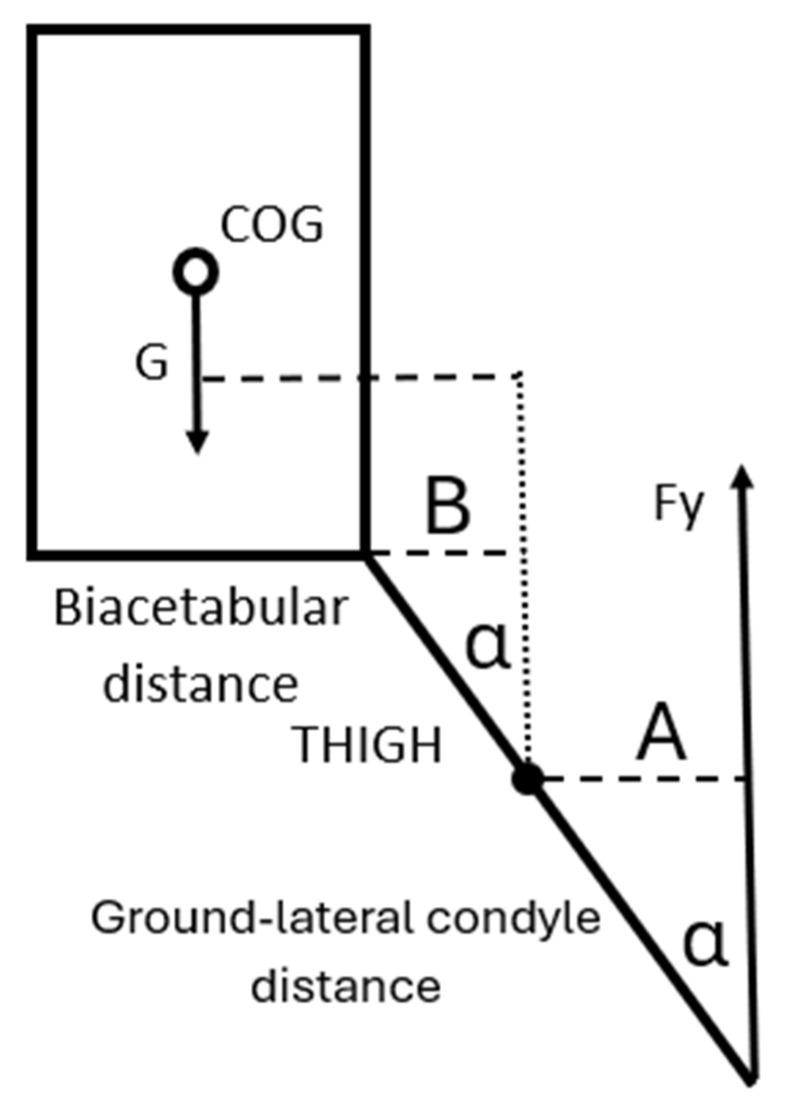
To determine the abduction torque of Fy for the knee, the moment arm is A = Ground-lateral condyle distance ▪ (sinα); the moment arm of G is biacetabular distance/2 + B = biacetabular distance/2 + THIGH ▪ (sinα).

We have created formulas to calculate the torques acting on the lateral condyle in a one-legged landing situation while assuming the sliding of the foot in the case of no traction, if the rotation around the lateral condyle is present as previously described in the mechanism above. The angle α represents the angle between the lower extremity and the vertical line (Figure 7). In the model, we hypothesize that the trunk is in a vertical position. We must emphasize the assumption that zero traction is critical to the model’s output, and can be regarded as a limitation. However, if the traction is greater than zero, the Fx component is also greater than zero, and consequently the tensile stress on the ACL will be smaller.

Torque generating abduction around the lateral condyle: (Fy ▪ lateral condyle moment arm) = (Fy ▪ Ground-lateral condyle distance ▪ (sinα)), where α is the angle of the shin measured from the vertical line.

Torque generating adduction around the lateral condyle: (ACL stress ▪ ACL-lateral condyle moment arm) + (MCL stress ▪ MCL-lateral condyle moment arm) + (patellar tendon stress ▪ patellar tendon-lateral condyle moment arm) + (semitendinosus stress ▪ semitendinosus-lateral condyle moment arm) + (bodyweight ▪ COG-lateral condyle moment arm)—where each moment arm reflects the appropriate force, respectively.

We intended to determine the angle of the shin measured from the vertical line (α) at which the ACL (and other components) would reach the point of a high risk of injury as the function of Fy. To calculate this angle, we have determined when the torques responsible for abduction are equal to the torques responsible for adduction around the lateral condyle. We have decided to use a conservative approach; therefore, we have used the maximum values that we found in the literature for the force-generating capabilities of the muscles for young healthy adults and the maximal stress the ligaments can withstand without damage (Table 1). We are aware that the model might overestimate the injury risk because of the conservative approach by not taking into account inter-individual variability.

**Figure 8:**
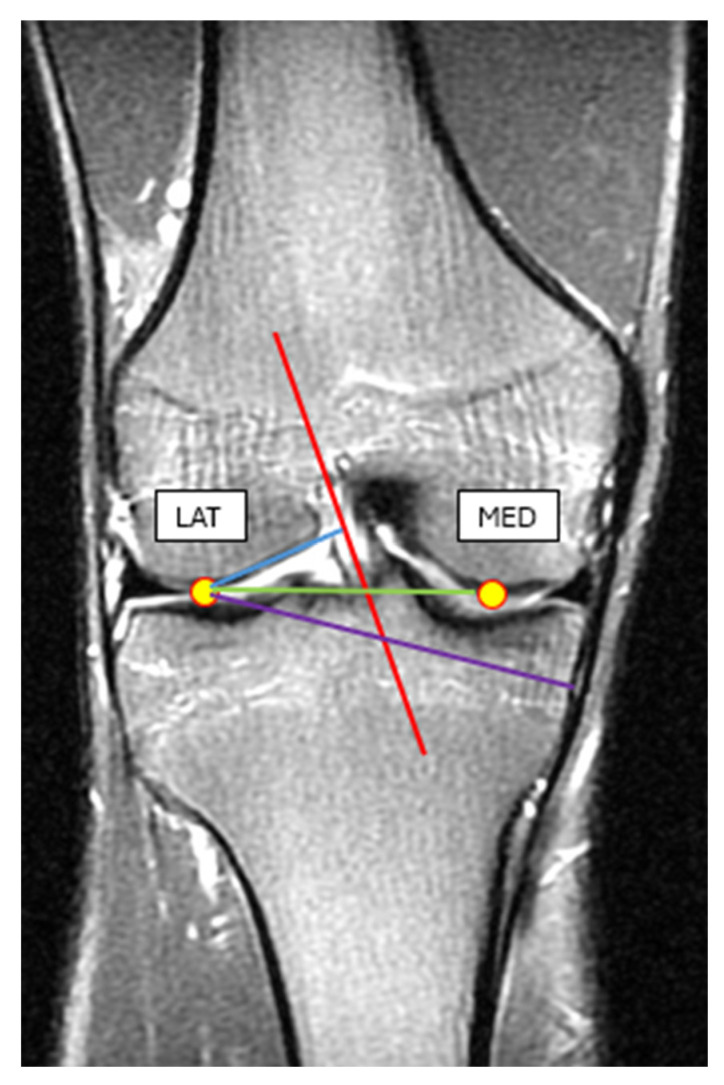
MRI image of the knee in an extended position. Blue line: the lateral condyle–ACL distance, purple line: lateral condyle–MCL distance, green line: lateral condyle–medial condyle distance.

To reach our goal, we have also collected MRI images and anthropometric data of 15 healthy male individuals with more than 10 years of organized sports background (age = 19–23 years, body height = 188.5 ± 8.6 m, body weight = 80.2 ± 6.8 kg). MRI measurements were taken from the individual’s dominant leg. In the MRI images of the knee, we have identified the lateral and the medial condyles and by using the MRI image software (RadiAnt DICOM Viewer 2024.2 version), we measured the lateral condyle–ACL distance, lateral condyle–MCL distance, and lateral condyle–medial condyle distance (Figure 8). For the required anthropometric data, we have measured the ground-lateral condyle and the lateral condyle–greater trochanter distance, respectively (Figure 8, Table 2).

**Figure 9:**
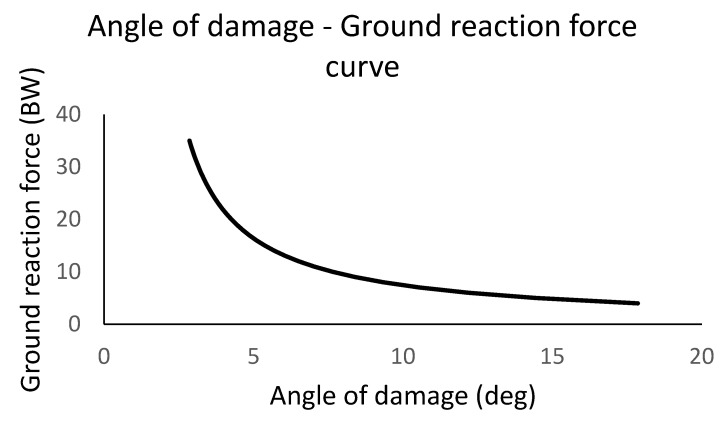
The graph represents the angles of the shin from the vertical line (angle of damage) at the initial contact at landing as the horizontal axis value, and the ground reaction force multiplied by body weight (m▪g) is being used as a unit for the vertical axis value. GRF forces were determined as a parameter to calculate the angle of damage values. The curve indicates that if the ground reaction force at a given landing angle is greater than the value of the curve, then based on the model the risk of injury in the ACL is significant.

Using the measured data and data obtained from previous research material, we have calculated the net torque acting on the lateral condyle in a one-legged landing as the function of the angle between the shin and the vertical line as described in the model. We hypothesize that if the torque originating from GRF resulting in abduction is greater than the torques that act as adductors, then the risk of an injury in one of the components that is responsible for the adduction is significant. Consequently, we have calculated the GRF as the function of the angle where the abduction and adduction torques are equal (angle of damage) by using the measured values in the formula below (our model is not gender-specific, but later for a more detailed model, gender differences should be taken into consideration):

Fy ▪ 0.59486 ▪ sin(α) = 2300 ▪ 0.02606 + 799 ▪ 0.0688 + 8000 ▪ 0.05846:2 + 1604 ▪ 0.0688 + 802 ▪ (0.12378 + 0.4618 ▪ sin(α)) where GRF, ACL, MCL, PT, SEMIT, COM torques acting on the lateral condyle were determined, respectively (all valuables use the same units (Nm) in the used equation; torque values were calculated using averages of previously obtained data). To calculate torques in the numerical equations, the values are Forces (N) multiplied by moment arms (m), respectively. The GRF-angle of the damage curve is visible in Figure 9. The curve was generated in Microsoft Excel through calculating the GRF values as the function of the angle values numerically using the formula introduced above applying 0.01 deg steps in the angles to calculate the points in the graph. If the GRF at a given landing angle is greater than the value on the graph, then based on the model the risk of injury is significant. In the graph, it is clearly visible that as the angle between the shin and the vertical line increases during landing, smaller GRF magnitudes can result in an ACL injury.

## 4. Discussion

By analyzing our results, we agree with numerous studies that knee valgus increases the risk of abduction torques and the risk of an ACL injury [10]. Our results are in concordance with Hewett et al. [8], as the model indicates that trunk position affects adduction torques, namely trunk lean angle influences the COG moment arm from the knee. We must emphasize that the landing posture we have discussed is highly unfortunate for the ACL. In normal circumstances, at landing, the significant GRF is dampened by the collective flexion of the hip–knee–ankle joints. However, if the knee is fully extended and the thigh is in an abducted position during landing, then the dampening effect in the knee and the hip is restricted. Furthermore, by using the model, we have identified for a given shin angle a minimal GRF value, and concluded that if this value is exceeded, the risk of injury in the ACL is substantial. We must emphasize that although in the literature ACL injury is associated with knee valgus, our hypothesis for the injury mechanism is in concordance with the model calculation of Hinckel et al., who concluded that if the knee is in an extended position, minimal changes in varus and consequently abduction angle will result in significant increase in stress on the ACL [41].

One notable factor to consider is that as the force-generating properties are significantly greater in the quadriceps—this is evident based on the thoroughly discussed H to Q strength ratio in the literature [42]—then the activation level of the quadriceps is a crucial factor. Any disturbance in the activation either as a result of a previous injury, delayed activation, disturbance in the proprioception, or fatigue either in the muscle cells or in the neural system will decrease the abovementioned compensation effect of the quadriceps, and consequently the load on the ACL will be greater [5,10].

We must emphasize that the time factor in obtaining a high quadriceps muscle activation level in our model at landing is crucial. Therefore, there is very limited time available for the quadriceps to develop the proper amount of tension (abrupt/ultrafast activation is needed) that could compensate for the torque of the GRF; consequently, minimal perturbation in the force-development parameters might result in the injury. This is in line with the observation that delayed-onset muscle soreness (DOMS), also theorized to be initiated by proprioceptive terminal Piezo2 channelopathy [32], alters the response to postural perturbations [43].

**Figure 10:**
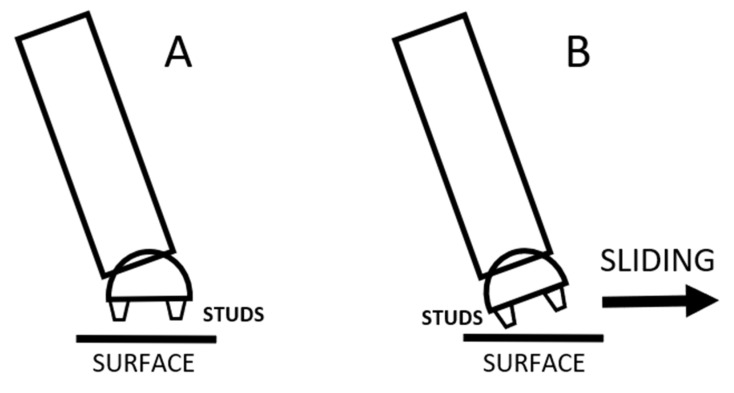
The shin and the foot of the athlete in the frontal plane in the moment of landing, also indicating the position of the studs. In (**A**), the foot is parallel with the surface and the studs provide adequate grip, while in (**B**), as the foot is not parallel with the surface, the possibility of sliding of the foot at landing increases.

We hypothesize that the traction parameters of the surface have extreme importance in this injury mechanism. If the surface is less slippery, the Fx component of the GRF will be greater and the net GRF torque acting on the knee will be smaller. Concerning the traction parameters while landing, the position of the ankle and the foot significantly affects the horizontal movement of the leg. Concerning soccer, the shoe is equipped with studs with the basic role to provide sufficient contact and traction with the surface. But if the landing occurs when the angle of the shin deviates from the vertical in the frontal plane, then contact and consequently traction might not be optimal. Two scenarios should be considered based on consultation with numerous professional soccer and handball coaches. If there is an angle in the shin from the vertical, as mentioned above, then one possibility is that the ankle is in a supinated position paired with dorsal flexion. This way the foot can be parallel with the surface at landing, and consequently the studs of the shoe can generate grip on the ground and traction will be sufficient (Figure 10A). The problem with this scenario is that because of the previously mentioned dorsal flexed position of the ankle at landing, the dampening effect of the ankle is limited; therefore, the GRF will be greater. Consequently, although Fx will be present, the vertical Fy component of the GRF will be extreme and the abduction torque on the lateral condyle of the knee will also be significant. No supination occurs in the ankle in the other scenario, and the foot is not parallel with the surface at contact. In this case the possibility of the sideways sliding movement of the foot at contact increases, as the studs are not able to provide sufficient grip and traction with the ground. Consequently, Fx will be significantly smaller or none, and in this case the abduction torque on the lateral condyle of the knee will also be significant (Figure 10B). The probability of occurrence of this scenario increases as the angle of the shin from the vertical observed from the frontal plane increases.

Moreover, while discussing the injury mechanism with professional soccer players, every individual highlighted that as the match progresses, moisture buildup inside the shoe at the heel is significant as the consequence of water on the field and the capillary effect of the socks. This phenomenon increases the medial-lateral (sideways) sliding effect of the heel inside the shoe. The structure of the soccer shoe is also not in favor of stabilizing the heel, as compared to a basketball shoe, therefore the shoe leaves the ankle open, providing minimal—if any—support for the ankle. This is necessary to enable extensive plantar–dorsal flexion, and also significant supination–pronation movement of the foot for kicking tasks, but results in minimal support for the ankle joint and for the heel. Consequently, a sideways slide of the heel inside the shoe can occur, even if the contact of the shoe with the ground using the studs provides adequate grip at landing. If a sideways slide of the heel occurs inside the shoe, then the effect is similar to the one discussed and represented above in Figure 10B.

Furthermore, it is worth mentioning that although the model did not count for the initial abduction angle between the thigh and shin [44], it can be hypothesized that if this abduction angle is greater than the phenomenon discussed above, then it would result in a greater risk of injury.

From a practical point of view and considering a preventive approach, the traction parameters of the surface should be increased based on our results. For example, the practice of creating wet grass turf should be avoided, and the shoes should be redesigned to minimize the slippage when the landing occurs in an ankle position described in Figure 10B.

When it comes to our primary damage working hypothesis, it is noteworthy that the Piezo2 ion channel is the principal mechanosensitive ion channel responsible for proprioception as was demonstrated by Nobel laureate Ardem Patapoutian [45]. In addition, Piezo2 is suggested to be the only ion channel capable of initiating an ultrafast long-range proton-based non-synaptic signaling within the nervous system; hence, they may be primarily responsible for ultradian sensing and responding to ultradian events [32]. The ultradian sensor detects ultradian events by initiating ultradian rhythm in humans. Ultradian rhythms are short biological cycles (less than 24 h), in contrast to the diurnal ones (24 h cycle). Hence, the acquired functional microdamage of this Piezo2 ion channel may lead to the aforementioned primary damage [32]. Moreover, the consequent loss of the proprioceptive protective function due to this primary damage may lead to resultant secondary harsher tissue damage of this neurocentric bi-phasic non-contact injury mechanism theory [32]. Accordingly, it has been proposed that over-excessive axial compression forces induce the preceding neuromuscular disruption by instigating indentation on Piezo2, leading to Piezo2 channelopathy, prior to non-contact ACL injury occurrence [11,14]. It is noteworthy that even bone bruises show on MRI images that non-contact ACL injury evolves only on a secondary locked subluxated position in the late phase and not as a result of the mechanism leading to ligament failure [46], likely indicative of the primary damage.

The slippery surface may not only increase axial compression forces and in response a point of attack of the resultant force in the medial proximal tibia during landing, but might induce over-excessive stretch (mimicking an over-excessive eccentric contraction) and resultant contribution to the aforementioned theorized microdamage of Piezo2 (failure to functionally activate abruptly/ultrafast). Correspondingly, this acquired Piezo2 channelopathy mechanism could not only explain the impaired response to perturbations, but also could explain that these non-contact injuries primarily happen in game situations when ultradian sensing may be overloaded due to strenuous and elevated acute stress situations. After all, this may be how the axial compression forces could induce Piezo2 channelopathy in encapsulated large fiber terminals of the medial part of the proximal tibia [14] and this might be how the preceding neuromuscular disruption could evolve not only by the instigated indentation, but excessive stretch of intrafusal Piezo2 on affected proprioceptive terminals, leading to Piezo2 channelopathies, prior to the non-contact ACL injury occurrence.

Indeed, a recent traumatic brain injury (TBI) study showed the critical contribution of PIEZO2 in the defensive arousal response (DAR) [47]. It is noteworthy that mild TBI is implicated to have an analogous bi-phasic non-contact injury mechanism on the periphery, like in the case of non-contact ACL injury and DOMS, where the primary damage could be an acquired proprioceptive neuron terminal Piezo2 channelopathy as well [32]. This DAR mechanism is essential for survival, and it is activated by a perceived threat and evoked by visual and auditory cues in the presence of motor abilities [47]. Moreover, DAR could be analogous to the acute stress response (ASR) that is part of the neurocentric non-contact ACL injury [11,14], and DOMS theory [32], and might often be induced during game situations. It is notable that a new preprint manuscript underpins the proton-based ultrafast matching/synchronization of the Piezo2-initiated eye–brain, auditory/vestibular–brain, and proprioceptive muscle–brain axes within the hippocampal hub [48], in line with the aforementioned DAR mechanism. Moreover, Pedemonte et al. showed earlier that sensory processing could be temporally organized by ultradian brain rhythms in addition to the temporary synchronization of the heart rate, medulla firing and the hippocampal theta rhythm under a homeostatic state [49,50]. Accordingly, it has been proposed that the beforementioned TBI genetic study may underscore Piezo2’s ultrafast ultradian sensory and ultradian rhythm generation function [51]. Consequently, acquired Piezo2 channelopathy may explain why DOMS alters the response to postural perturbations [45] and significantly increases the medium latency response of the stretch reflex [52]. As a result, Piezo2 channelopathy may impair the abrupt/ultrafast activation of the medium latency response of the stretch reflex (that could be the static phase firing encoding), leading to impaired ultradian sensory and ultradian rhythm generation function. In support, another recent research paper demonstrated that the very fast activation of rapidly adapting currents was reduced in neurons from *Piezo2*^+/−^ and *Piezo2*^−/−^ embryos, as opposed to *Piezo2*^+/+^ neurons [53]. Moreover, the same study showed that the inactivation kinetics of all mechanically gated currents were also slowed by 2.5 to 5-fold from *Piezo2*^+/−^ and *Piezo2*^−/−^ neurons, in contrast to the ones from *Piezo2*^+/+^ neurons [53]. These findings may not only support the theorized ultrafast sensory function of Piezo2 [32], but also underpin the principality of Piezo2 in the mechanotransduction of proprioception, as Ardem Patapoutian and his team reported [45]. However, it is important to highlight that other ion channels also contribute to proprioception, but the current authors propose that Piezo2 is the only one providing the fine/ultrafast control of proprioception as part of ultradian sensing, reflected in its aforementioned principality.

Therefore, the Piezo2 functions under DAR/ASR are analogous to the proposed underlying Piezo2-initiated proton-based ultrafast ultradian hippocampal backbone of brain axes, including the bone–brain and muscle–brain axes in the current scenario that are temporarily organized to hippocampal theta rhythm. However, Piezo2 channelopathy may impair this finely regulated Piezo2-initiated ultrafast ultradian hippocampal organization due to over-excessive axial compression forces. As a result, this acquired Piezo2 channelopathy could induce a somatosensory switch/miswiring and resultant sensory mismatch, exaggerated quadriceps contractions and delayed static phase firing encoding that puts greater load on the ACL [11,14]. Furthermore, this microdamage of Piezo2 also lessens the ability to react to ultradian events, especially perturbations, e.g., slippery surface and sliding, hence making the ACL more prone to injury. Accordingly, abrupt/ultrafast reactiveness to perturbations of Piezo2 may come from quantum mechanical properties, where Piezo2 initiates the ultrafast proton-based long-range signaling, but acquired Piezo2 channelopathy may impair this ultrafast capability, leading to increased injury risk [32].

We must emphasize that although we have found studies comparing the effect of different surfaces on ACL injury occurrence [54,55], to our knowledge no prior model has investigated the differences in friction—neither considering static (μ_0_), nor kinetic/dynamic (μ)-parameters on one given surface regarding ACL injury. Moreover, we have not found any research where field friction parameters and neuromechanical dysfunction as a result of the theoretical Piezo2 microdamage have been previously linked.

## 5. Limitations

We must highlight that the aim of the manuscript was to address a complex biomechanical-neurocentric mechanism while trying to introduce a simplified model for which several values were missing in the literature, therefore there are numerous limitations arising concerning the study of the current manuscript.

To acquire proper data for the model, we relied on data from previous studies concerning muscle force values and ligament tensile strength values. Moreover, to obtain generalizable anthropometric parameters, the sample size of 15 is limited, but the focus was not on building an anthropometric database, but to acquire minimal data with which we can introduce the model. It is also worth mentioning that in order to introduce the model, we had to rely on data from the literature, therefore we may have overestimated torques and biased the analysis towards worst-case scenarios. We are aware that the model was generated based on data from only men. However, by thoroughly examining the literature, in many cases we could only find available tensile strength values for men. Therefore, it is a future goal to modify the model using values appropriate for women as well.

We must also highlight that we have used video recordings of non-contact ACL injuries during match situations for the preliminary idea for this paper. We did not find any reliable data in the literature about the ACL injury occurrence regarding match vs. training situations, but before the study, we consulted with several soccer and handball coaches and physiotherapy experts who unanimously stated that in their experience, ACL injuries are more frequently observed in game situations.

We must note that the injury mechanism that we have introduced uses a 2-dimensional model and therefore does not consider the probability of tibial rotation and anterior translation occurrence or H/Q ratio which are also key components of ACL loading. Although the model is a 2-dimensional simplification, if we were to take into account the rotation discussed in detail in the previous literature of the tibia and the femur in the opposite direction (that would occur—considering this posture—around the contact point between the lateral condyles), then in our opinion, even greater stress would act on the ACL. However, a more complex 3-dimensional model should be introduced in the future in order to calculate the effect of rotation. We must also emphasize that the nature of modeling is the simplification of a very complex phenomenon, and we needed this constraint to introduce this model.

Although we introduce the mechanism of slippery surface into the model, we do presume that the coefficient of friction (μ) is zero. We are aware that in this conservative model the assumption of zero coefficient is an extreme situation, but if the traction is greater than zero, the Fx component is also greater than zero, and consequently the stress on the ACL will be smaller. Therefore, further modeling is necessary to build in the coefficient of friction (μ) as a new independent variable into the model.

In addition, kinematic or video-based measurements of joint angles at landing in the scenario that we have introduced would considerably strengthen the validity of the model.

We are aware that no empirical validation of the model is presented (e.g., comparison with force plates or 3D motion-capture data), which limits the credibility of the simulations, but for us it was not achievable to recruit participants and increase the load of their ACL in a laboratory environment until it completely tears. Therefore, as we were not able to use 3-dimensional movement analysis data during an ACL injury situation, our model is not supported by this technology. In our opinion, one way to enhance the model’s credibility and applicability in the future is to use a movement analysis system on previous video recordings of ACL injuries. As matches are recorded with more than one camera, using a movement analysis system like SIMI Motion, which uses the contours of the athletes to generate a 3-dimensional model, it would be possible to apply an inverse dynamic model even in game situations.

## 6. Conclusions

In this paper we intended to investigate the effect of different factors on ACL load during a landing phase of a vertical jump. We have concluded that in an unfortunate situation when the knee is in an extended position during the landing phase of a vertical jump, if the angle of the shin measured from the vertical is greater, then the stress is greater in the ACL. Moreover, the surface traction parameters also have an effect, such as the slippery soccer field and handball court in game situations during landing; therefore, the stress on the ACL is going to be greater as well. We must consider one more factor in this injury mechanism, namely if the proprioceptive neural control of the affected muscles is disrupted prior to the overload and moment of injury, then the compensatory mechanisms originating from the proper abrupt/ultrafast activation of the muscles cannot decrease the stress on the ACL, leading to increased injury risk of the ACL. Finally, we applied a Piezo2 ion channel-related molecular working hypothesis of this preceding proprioceptive microdamage-derived disruption of the neuromuscular system that is not only in line with earlier theory [11], but with the observation of Boden and Sheenan in reference to non-contact ACL injury [13].

## Figures and Tables

**Table 1:** Data of mechanical properties of various components from the previous literature for the model. For the data, a source was used with samples consisting of young healthy adults.

Variables FROM Literature *	ACL Max Stress	MCL Max Stress	Quadriceps Max Force	Gastrocnemius Med. Max Force	Semitend + Semimembr Max Force	Bi-Acetabular Distance/2
	2300 N	799 N	8000 N	931 N	2 BW	123.78 mm

* Variables from previous research articles [29,35,37,38,39,40].

**Table 2:** Average and SD of measured variables for the athletes. MRI images were used to determine the values for the internal section of the knee.

Measured Variables (Distances)	Lateral Condyle-ACL (mm)	Lateral Condyle-MCL (mm)	Lateral Condyle-Medial Condyle (mm)	Ground-Lateral Condyle (mm)	Lateral Condyle-Greater Trochanter (mm)
Average	26.06	68.8	58.46	594.86	461.8
SD	1.7	3.27	2.82	13.89	9.33

## Data Availability

The data presented in this study are available on request from the corresponding author due to containing information that could compromise the privacy of research participants.
